# Project PAVE (Personality And Vision Experimentation): role of personal and interpersonal resilience in the perception of emotional facial expression

**DOI:** 10.3389/fnhum.2014.00602

**Published:** 2014-08-13

**Authors:** Michal Tanzer, Golan Shahar, Galia Avidan

**Affiliations:** Department of Psychology, Ben-Gurion University of the NegevBeer Sheva, Israel

**Keywords:** angry expression, happy expression, general self-efficacy, perceived social support, biased emotion recognition

## Abstract

The aim of the proposed theoretical model is to illuminate personal and interpersonal resilience by drawing from the field of emotional face perception. We suggest that perception/recognition of emotional facial expressions serves as a central link between subjective, self-related processes and the social context. Emotional face perception constitutes a salient social cue underlying interpersonal communication and behavior. Because problems in communication and interpersonal behavior underlie most, if not all, forms of psychopathology, it follows that perception/recognition of emotional facial expressions impacts psychopathology. The ability to accurately interpret one’s facial expression is crucial in subsequently deciding on an appropriate course of action. However, perception in general, and of emotional facial expressions in particular, is highly influenced by individuals’ personality and the self-concept. Herein we briefly outline well-established theories of personal and interpersonal resilience and link them to the neuro-cognitive basis of face perception. We then describe the findings of our ongoing program of research linking two well-established resilience factors, general self-efficacy (GSE) and perceived social support (PSS), with face perception. We conclude by pointing out avenues for future research focusing on possible genetic markers and patterns of brain connectivity associated with the proposed model. Implications of our integrative model to psychotherapy are discussed.

The notion that individuals and social context actively shape each other, evident in numerous conceptual perspectives, is represented in Albert Bandura’s seminal principle of *reciprocal determinism* (Bandura, 1978; see also Shahar, 2006 for review of such action models in clinical psychology). Herein we extend this notion by proposing an integrative model that incorporates research on perception and recognition of emotional facial expressions. Specifically, we posit that biased emotional face perception and its relation to individuals’ personality and self- concepts may explain vulnerability to, and resilience in the face of, a host of psychological difficulties. We begin by providing a brief overview of the well-established concepts that contributed to our overarching model. We then describe findings emanating from our ongoing program of research entitled **Project PAVE** (**P**ersonality **A**nd **V**ision **E**xperimentation) which link personal and interpersonal resilience and perception of emotional facial expressions. We conclude by noting avenues for future research focused on possible genetic markers and patterns of brain connectivity associated with the proposed model, as well as implications for psychotherapy.

## Perception of facial expression and its role in vulnerability to, and resilience in the face of, psychopathology

**Figure 1:**
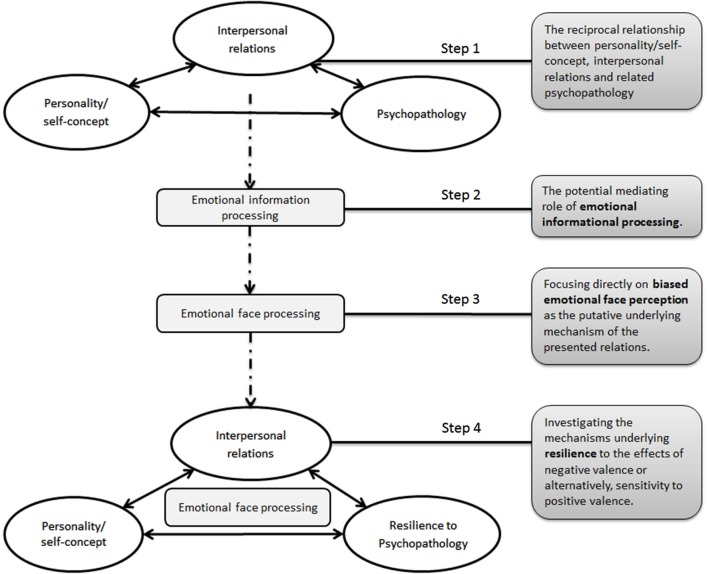
**The proposed theoretical model suggesting biased emotional face perception as the putative underlying mechanism of the reciprocal connections between personality/self-concept, interpersonal relations and resilience to psychopathology**. See the boxes in the right panel for the related steps that led us to develop this overarching model.

As presented in Figure 1 (Step 1), mounting evidence in social, developmental, and clinical psychology, inspired by Bandura’s principle, highlight the active role of individuals in shaping their own environment, consequently, affecting interpersonal relations, risk to psychopathologies or their self-concept (Lerner, 1982; Swann, 1983, 1990; Buss, 1987; Hammen, 1991; Joiner, 1994; Wachtel, 1994; for review, see Shahar, 2006). For example, depressed or self-critical individuals may generate interpersonal aversive circumstances that eventually maintain or elicit their depressive state and/or their self-criticism (Joiner, 1994; Mongrain, 1998; Joiner et al., 1999; Zuroff and Duncan, 1999; Priel and Shahar, 2000; Shahar and Priel, 2003; Blatt and Shahar, 2004; Shahar et al., 2004; Bareket-Bojmel and Shahar, 2011; Shahar and STREALTH LAB, in press).

But what is the mechanism underlying these findings? According to some social theories, individuals form their self-concept, at least in part, based on the ways others observe them and relate to them (e.g., the looking glass; Cooley, 1902). A similar notion was also postulated by the well-known psychoanalyst Winnicott in his theory regarding “mirroring” (1971), according to which infants form their sense of self by mentally absorbing their mother’s facial expression as she attends to them. Relatedly, according to Swann’s self-verification theory, people are motivated to search for evidence confirming their self-concept, and this motivation influences perceptual information processing (Snyder and Swann, 1978; Murray et al., 2000) as well as social interactions (Swann et al., 1989, 1994). Specifically, depressed individuals are more prone to interactions with partners who perceived them unfavorably and were indeed more alienated and rejected than non-depressed individuals (Swann et al., 1992).

Another approach for understanding this vicious cycle comes from Beck’s cognitive model, stating that depressed individuals are likely to process information in a dysfunctional manner and this biased acquisition and processing style contribute to the maintenance of their psychopathology (Beck, 1967, 2008). Studies supporting this notion stress the causal role of biased attention in increased emotional vulnerability and investigate how interventions that modulate biased processing affect psychopathological disorders (for a related review see Mathews and MacLeod, 2002; Browning et al., 2010; Hakamata et al., 2010; Wells et al., 2010, and special section on cognitive bias modification in The Journal of Abnormal Psychology, Koster et al., 2009, but see Hallion and Ruscio, 2011). For example, the induction of an attentional bias by manipulating the location of threatening/neutral words prior to the presentation of to be detected probes using the dot probe paradigm, modified individuals’ response time and consequently affected their mood during a standardized stress manipulation. Specifically, the group that was biased toward threat by being presented with threatening words prior to the probe, exhibited a greater increase in negative mood during the following stress task, compared to the group presented with neutral words (MacLeod et al., 2002).

Thus, previous studies imply that biased information processing and specifically, social-emotional information, may play a primary role in the development and maintenance of psychopathology, (Beck, 1967; Mathews and MacLeod, 2005; Bar-Haim et al., 2007; Clark et al., 2009; Disner et al., 2011; Roiser et al., 2012)—in turn affecting interpersonal relations (Swann et al., 1992; Shahar, 2006). This process is illustrated in Step 2 of Figure 1.

Previous studies have attested to segments of the processes presented in Figure 1. For example, anxiety has been associated with the tendency to attend to threatening information [e.g., the emotional Stroop (Stroop, 1935), the dot probe task (MacLeod et al., 1986) and the emotional spatial cuing paradigm (Fox et al., 2001), for further elaborations on these tasks see reviews by Bar-Haim et al., 2007; Cisler and Koster, 2010]. Using the dot probe paradigm (see description above), it has been demonstrated that individuals with a general anxiety disorder are faster to respond to probes replacing threat words, than neutral words, as compared to controls (e.g., MacLeod et al., 1986; Mogg et al., 1992). Similarly, depression was associated with a bias toward negative congruent information, mostly due to a difficulty in disengaging from information with a negative valence (for reviews see Gotlib et al., 2004; Mathews and MacLeod, 2005; Clark et al., 2009; Gotlib and Joormann, 2010; Disner et al., 2011; Roiser et al., 2012). Relatedly, in the emotional Stroop task, in which participants’ response time to name the color of an emotional written word indicates their ability to disengage from the emotional context, depressed patients were slower to name the color of negative emotional words, compared to non-depressed controls (Gotlib and McCann, 1984; Broomfield et al., 2007).

In relation to the above, Roiser et al. (2012) proposed a cognitive neuropsychological approach for the understanding and treatment of depression. This model is based on a presumed casual chain linking negative information processing (e.g., biased emotional perception, attention and memory) to the development of symptoms of depression. Presumably, such a cognitive bias is affected by alterations in biological factors (e.g., monoamine transmission via different brain circuits involved in affective regulation and processing) and their interactions with both environmental and genetic factors (see the detailed model in Roiser et al., 2012). Importantly, this model was based on results obtained from a longitudinal design (Forbes et al., 2007) as well as on studies conducted with individuals at risk for developing depression, or on ones that recover from it (see Roiser et al., 2012).

Within this general theoretical template, our own particular contribution lies in the focus on biased processing of emotional facial expressions as depicted in Step 3 of Figure 1.

Given its unique evolutionary and social significance, face perception is probably the most multifaceted visual perceptual skill in humans. In addition to invariant information such as identity and gender, faces convey a large amount of subtle, variant, changeable information such as age (Ishai, 2008), expressions (Fox et al., 2000), intentions (van’t Wout and Sanfey, 2008) and mood (Adolphs, 2003) upon which human observers rely for social interaction and communication. A wealth of behavioral literature posits that this efficient and multifaceted processing of faces is accomplished *in a qualitatively different* fashion compared to the processing of other object categories. Specifically, deriving a rapid and accurate representation of the face requires a disproportionate reliance on the configuration of the physical features of the face relative to that required for non-face object recognition (Behrmann et al., in press). This *holistic processing* is considered a hallmark of face perception (Farah et al., 1995; Richler et al., 2011; Behrmann et al., in press; DeGutis et al., 2013). Neuroimaging studies in humans collectively point to a number of “core regions” that show selective responses associated with the visual invariant, as well as variant, properties of faces. Additionally, there are a number of regions outside the occipito-temporal cortex that constitute an “extended” face recognition system with unique roles in processing high-level attributes of face perception such as memory and emotion (Haxby et al., 2000; Gobbini and Haxby, 2007; Ishai, 2008).

Of all the different types of information embedded in the face, facial expressions are of most relevance to the present investigation. Emotional face perception constitutes a key mechanism for social communication which is crucial for forming appropriate actions during social interactions (Öhman and Mineka, 2001; Haxby et al., 2002; Russell et al., 2003). Individuals’ facial expressions allude to the expresser emotional state and may elicit a similar response in the observer (Haxby et al., 2002). The preference to look at face-like stimuli can be observed in newborns (Johnson et al., 1991), and first signs of facial expression recognition abilities are witnessed during the first year of life (Walker-Andrews, 1997; De Haan and Nelson, 1998; Farroni et al., 2007). Moreover, the process of recognizing an emotion from a face in order to produce a conceptual knowledge of this expression was suggested to involve areas in the core and extended systems via their anatomical and functional connections (Adolphs, 2002).

Furthermore, and most pertinent to our proposed model, psychopathological disorders were shown to be closely associated with biased processing of emotional face stimuli (see Mathews and MacLeod, 2005; Cisler and Koster, 2010; Yiend, 2010, for reviews). For example, individuals suffering from comorbid anxiety and depression recognized angry expressions better than happy and neutral expressions, a pattern that is reversed compared to controls (Gilboa-Schechtman et al., 2002). Additionally, Jermann et al. (2008) reported a positive correlation between depressive symptoms and the conscious recollection of sad expressions. Moreover, socially phobic patients better recalled faces that they judged as “critical” at the learning phase, while non-anxious controls performed better with faces that were judged as “safe” (Lundh and Öst, 1996; Coles and Heimberg, 2005).

The notion of biased attention toward recognition of facial expressions is also related to the idea that individuals’ thoughts and feelings about themselves are closely related to the way in which they believe others perceive them (Cooley, 1902; Sullivan, 1953; Shraugher and Schoeneman, 1979). Moreover, the way individuals perceive themselves affect the way they perceive others (Swann, 1983, 1990; Leary, 1990). This notion is well captured in the seminal quote by Merleau-Ponty (1964) “*I begin to live my intentions in the facial expressions of the other and likewise begin to live the other’s volitions in my own gestures” (p. 119*). Thus, through the prism of emotional face perception which is shaped by one’s own self-views, individuals interpret their social environments, and this subjective interpretation, may in turn affect psychopathology and project back on their self-perception.

But what about resilience to psychopathology? Individuals have the ability to adapt, cope and maintain a stable equilibrium in the face of life stressors (Rutter, 1985; Richardson, 2002; Bonanno, 2004; Shahar et al., 2012). Yet, the question of why some people are more emotionally resilient than others still awaits an answer. We suggest that the relation between resilience factors such as personality traits or social variables, and processing of emotional face perception may be informative for understanding risk/resilience to psychopathology in terms of prevention: by investigating what makes some people more immune to the effects of negative valence or alternatively, more subjected to positive valence, we may be able to identify those individuals who are most vulnerable to adverse circumstances (Hauser et al., 2006; Shahar, 2012). Step 4 of Figure 1 depicts our full-fledged model.

A number of factors have been associated with resilience, among them having high self-esteem or self-efficacy (Garmezy, 1991; Werner and Smith, 1992; Rutter, 1993; Masten, 1994), having emotional stability, extraversion or agreeableness (Friborg et al., 2005) and reporting elevated levels of perceived social support (PSS; Cohen and Wills, 1985; Kessler et al., 1985; Cohen et al., 2000; Cohen, 2004; Uchino, 2006; Lakey and Orehek, 2011). These factors were shown to contribute to positive outcomes and protect against negative ones. For example, social support has been shown to protect against a wide variety of adverse outcomes including depression (Lakey and Cronin, 2008), post-traumatic stress disorder (Brewin et al., 2000), and physical illness (Uchino, 2006) and to promote positive consequences such as self-care (Graven and Grant, 2014), coping strategies (Cohen and Wills, 1985; Davis and Swan, 1999; Wills and Fegan, 2001) self-control (Wills and Bantum, 2012) and optimism (Karademas, 2006). Importantly, there is almost no research on the possible underlying mechanisms mediating these effects particularly from the neuro-cognitive perspective, let alone focusing on face perception.

## Project PAVE

Project PAVE was launched in order to examine our proposed link between vulnerability/resilience, emotional face perception, and self/social functioning. In the following sections we will describe the findings emanating from this project and note some future directions and implications.

First, we examined the associations between general self-efficacy (GSE), a central dimension of personal resilience pertaining to individuals’ positive beliefs about their own capabilities (Bandura, 1997). We hypothesized that happy facial expression may signal approval by others, which should be congruent with the preceptor’s high self-worth. Thus, we predicted that GSE would be positively correlated with accurate recognition of happy facial expression.

**Figure 2:**
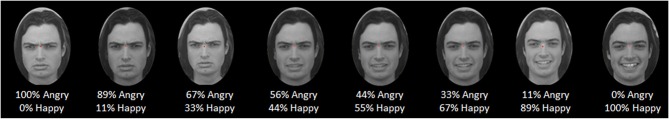
**Example of morph stimuli used in the experiments**. The original stimuli (AM01) were taken from the KDEF database (Lundqvist et al., 1998). In this example stimuli are comprised from angry and happy faces morphed together to create a continuum of blending.

To test our hypotheses, we used a morph technique that merged between two emotional stimuli to create a new image containing a specified percentage from each of the original stimuli (see Figure 2). This method enabled us to assess both accuracy and bias depending on the morph level of the dominant expression. Participants (*n* = 70) were asked to classify the expression presented in each trial. Accuracy was determined by the dominant expression within each morph blend. Prior to the behavioral task, participants completed a battery of questionnaires assessing their self-efficacy and depressive symptoms. As predicted, and even after controlling for depressive symptoms (in this, as well as in all other studies described below), individuals with high self-efficacy showed a specific bias towards recognition of happy facial expressions. We interpreted this effect as a way to maintain and form affirming relations, which may serve as a protective factor during stress (Tanzer et al., 2013a).

Next, we hypothesized that happy facial expression would be better memorized compared to angry expressions, as the former may serve as a potential shelter, one could lean on and recall in a time of need. Thus we conducted another study in which participants (*n* = 92) were asked to memorize faces portraying happy/angry expressions and then (after a short interval) to recall which face was previously presented and retrieve the portrayed expression. As expected, GSE was positively correlated with better identity recognition for faces portraying a happy expression during the learning phase and with the tendency to recall the learned expression as happy. Taken together, our findings suggest that individuals with high GSE are tuned, in terms of ***both*** recognition and memory, to “happy others”, possibly as a way of self-verification of their own positive self-views. This self-efficacious prism, through which one interprets his/her surrounding, may reduce stress and protect against potential hazards, consequently minimizing the risk for psychopathology (Tanzer et al., 2013a).

In our next line of studies we sought to examine other protective factors that are more related to the social context. Inspired by theories linking cognitive processes to interpersonal relationships (Leary, 1990, 2005; Pickett et al., 2004; Pickett and Gardner, 2005), we focused on PSS. PSS refers to the interpersonal network of resources that is available to individuals to provide help during time of need (Cohen and Wills, 1985; Lakey and Cronin, 2008; Lakey and Orehek, 2011). Based on the known role of PSS as a main protective factor against a wide range of negative life events or as a stress buffer minimizing their aversive outcomes (e.g., Cohen and Wills, 1985; Theran et al., 2006; Lakey and Cronin, 2008; Shahar et al., 2009; Lakey and Orehek, 2011), we predicted that it would be negatively associated with recognition of an angry expression, as the latter is a sign of threat one should avoid. Using the morph paradigm again, we now morphed between angry and neutral facial expressions and indeed found that individuals (*n* = 71) with elevated levels of PSS were less accurate in recognizing angry facial expressions (Tanzer et al., Submitted). Thus, positive PSS emerged as a protective factor that enables individuals to monitor their environments and overlook angry facial expressions, arguably being more open to positive and rewarding exchanges.

We also examined the impact of PSS on emotional face processing in a stressful situation by a failure/success manipulation (for details regarding the manipulation see Mendelson and Gruen, 2005; Tanzer et al., 2013b). Participants (*n* = 142) first filled questionnaires assessing their PSS and depressive symptoms and were then randomly allocated to a failure or a success condition, and accordingly were lead to believe they either failed or succeed at the Raven intelligence test (Raven et al., 1985). We hypothesized that PSS would act as a protective shield against hazards (e.g., an angry facial expression) in a time of need (e.g., the failure condition). Following the failure/success manipulation, they participated in the morph experiment that enabled assessing the accuracy and bias involved in recognition of emotional facial expressions (Figure 2). As expected, we found that in the failure group (i.e., where individuals were bogusly believed they failed an intelligence test alluding to their self-worth), participants with elevated levels of PSS, as compared to those with low levels of PSS, were less accurate in recognizing angry facial expression, possibly as a way to maintain their self-worth during a time of need (Tanzer et al., 2013b).

In a similar fashion, we continued our investigation and examined how induced social support interacts with individuals’ self-worth (i.e., GSE) in relation to recognition of an angry facial expression. Participants first completed questionnaires assessing their GSE, PSS and depressive symptoms (*n* = 54). They then took part in an imagery task, where they were asked to visualize a close partner or someone else who betrayed them in a time of need. Following this manipulation, they participated in the morph experiment. We predicted that both elements (i.e., positive support and elevated levels of GSE) would act synergistically to produce a bias against negative social cues (i.e., an angry facial expression). Such an intriguing interaction was indeed found and interpreted as a “protective shield” enabling individuals to monitor their surroundings in order to avoid recognition of angry expressions which consequently improve their well-being (Tanzer et al., Submitted).

## Conclusions, future directions, and implications

Taken together, these biases towards positive (e.g., happy expressions) facial expressions or against negative ones (e.g., angry expressions) may suggest biased emotional face processing as an underlying mechanism of the chain that leads from personality/self-concepts or interpersonal relations to risk/resilience to psychopathology. Protective factors (e.g., GSE and PSS), may serve as a “narrow” adaptive prism through which one interprets his/her surroundings. This biased perception, may consequently lead to selective attention to, or dismissal of, specific aspects of the environment, which eventually generate benevolent effects and reduce maladaptive ones. Whereas research on biased face processing in clinical populations has developed tremendously in the past decade (e.g., Mathews and MacLeod, 2005; Cisler and Koster, 2010; Yiend, 2010, for reviews), research on individual differences within the non-clinical populations is still in its infancy, and we suggest that focusing on the latter would open up an important avenue for better understanding of human behavior that in turn, may promote psychotherapy interventions.

Our suggested model emanated from different theories in diverse subfields of psychology (i.e., clinical, social and cognitive) and neuroscience. Thus, we were inspired from Bandura (1978) on reciprocal determinism and the perspective of action theory that stresses individuals’ role in actively shaping their own environment (Lerner, 1982; Brandstadter, 1998; Shahar, 2006). Additionally, we built upon Winnicott’s notion of the mirroring role of the mother as a vehicle for self-knowledge (Winnicott, 1971; see also Shahar and STREALTH LAB, in press), on social-clinical theories which aimed to explain how individuals construct self-views (e.g., the looking glass; Cooley, 1902), and how these self-views affect individuals’ perception [self-verification theory (Swann, 1983, 1990)]. Moreover, we were influenced by theories on biased cognition such as Beck’s notion on individuals’ dysfunctional schemes and its effect on information processing. Finally, we were inspired by our vast interest in face processing, in relation to cognitive and developmental aspects (Behrmann and Avidan, 2005; Behrmann et al., in press; Avidan and Behrmann, 2014). As is evident, even when designing the most “basic” cognitive paradigm, one should bear in mind the existence of individual differences and the interplay between individuals’ self and their outer subjective surrounding and these factors should be taken into account.

Our theoretical model alludes to neural mechanisms that may be involved in emotional face perception. While an extensive review of the vast literature on the neural basis of face perception lies outside the scope of this brief article (see Haxby et al., 2000; Gobbini and Haxby, 2007; Ishai, 2008; Rossion, 2014), we wish to point out the importance of focusing on the amygdala, known for its role in emotional face processing and its vast direct and indirect connections to cortical and subcortical structures, thus making it an important neural “hub” (LeDoux, 2000; Davis and Whalen, 2001). Specifically, it has been suggested that regulation of emotional stimuli may be accomplished by the reciprocal connections between the amygdala and orbital and ventro-medial prefrontal cortex (Adolphs, 2002; Vuilleumier, 2005). This coupling between the amygdala and prefrontal areas was in the focus of numerous studies, implicating its association with genetic individual differences [(i.e., genetic polymorphism), Hariri et al., 2002] and more specifically with allelic variation in the promotor region of the serotonin transporter gene (5-HTTLPR). For example, carriers of the s-allele, compared with the l-allele, of 5-HTTLPR showed elevated hemodynamic response to fearful expressions during fMRI scans (Hariri et al., 2002), which was associated with reduced coupling between the amygdala and the subgenual cingulate gyrus (Pezawas et al., 2005). Interestingly, an attentional bias toward happy facial expressions was associated with carrying of the “l” allele (Pérez-Edgar et al., 2010), thus possibly implicating this genetic variable as a potential protective factor against stressful life events (Fox et al., 2009).

Evidence more pertinent to our presented model and to the suggested future directions comes from studies that reported that the strength of the functional connections (as assessed with fMRI) between the amygdala and medial prefrontal areas was associated with the size of one’s social network (Bickart et al., 2012), as well as to diverse psychopathologies (e.g., anxiety: Kim et al., 2011a,b). Moreover, amygdala activation in response to happy facial expression was associated with the personality trait extraversion (Canli et al., 2002; Canli, 2004), that might have some associations with generalized self-efficacy. Furthermore, PSS was found to moderate the relation between amygdala activity in response to fearful and angry facial expressions and anxiety trait, such that only low PSS predicted the relation between amygdala activity and anxiety trait (Hyde et al., 2011).

Taken together, these different findings call for future studies that will enable their integration into a single comprehensive framework using diverse methodologies to measure functional signal in face related regions and the connectivity between these regions, as well as genetic, self and face processing measures. We hypothesize that individual differences in variables associated with self-concept will manifest in cognitive processing biases that would be related to gene polymorphism accompanied by variations in the coupling of amygdala and frontal areas. Accordingly, resilient individuals will show lower amygdala reactivity to angry faces, and this reactivity would be due to enhanced suppression from frontal areas.

Another related brain region that is considered part of the extended face processing network is the insula, known for its involvement in affective processing (Adolphs, 2002) and empathy (Wicker et al., 2003; Adolphs, 2009; Singer and Lamm, 2009). Consistently with this account, the abilities to recognize and experience facial expressions (specifically disgust) are impaired in individuals with bilateral lesions in the insular cortex (Calder et al., 2000; Adolphs et al., 2003). In addition to these roles, that may be mediated by the connectivity of the insular cortex to the amygdala, this region is also considered part of the visceral somatosensory cortex and hence may be involved in modulating introspective information (Craig, 2002, 2008) as well as mediating responses to aversive stimuli (Phillips et al., 2003a). Thus, in light of our findings, and emanating from the notion that self-perception affects how individuals modulate their outer surrounding, future studies linking the insula activation and functional connectivity during emotional face recognition and its associations with self/social variables are warranted. Importantly, previous findings already allude to such an association; for example, insula activation during emotional recognition was associated with trait anxiety (Stein et al., 2007), social phobia (Gentili et al., 2008), schizophrenia and affective disorders (for review see Phillips et al., 2003b).

Moreover, future longitudinal studies should enable the construction of a more cohesive map of the relations in our proposed model. Such a line of inquiry is also expected to illuminate other alternative explanations, for example that biased perception may serve as a consequence factor (Koster et al., 2009) being influenced by either/both psychopathology and/or resilience (MacLeod et al., 2002; Mathews and MacLeod, 2005; Yiend, 2010). We stress that even though all of our studies were conducted on a non-clinical population and we controlled for depressive symptoms (Tanzer et al., 2013a,b, Submitted), we cannot completely rule out other probable explanations such as the possibility that previous psychopathological conditions (e.g., anxiety or affective disorders) might have accounted for some of the bias found in our results.

Also, individuals’ past experiences and exposure to their caregivers’ facial expressions might not only influence how these individuals form their sense of self, but also the saliency of these expressions later on. For example, it has been demonstrated that maltreated children directed their attention away from angry faces, as compared to controls, and interestingly, this bias to avoid threatening stimuli was dependent on the severity of the physical abuse they suffered from (Pine et al., 2005). Also, as suggested above, future studies focusing on genetic markers and their interaction with self-variables in association with biased face processing, may shed more light on other possible explanations emanated from the nurture vs. nature problem (i.e., consequences vs. predispositions). Nevertheless, our experimental-manipulation alludes to the suggested interpretations that self/social variables serve as predispositions that may lead to a cognitive bias for emotional face perception (i.e., consequence) which may affect risk/resilience to psychopathology and not vice versa. Moreover, previous studies that examined these associations and explored psychological interventions to alter biased processing, found supportive evidence for such a causal link among healthy populations (Mathews and MacLeod, 2002; MacLeod et al., 2002; Browning et al., 2007, 2010; Murphy et al., 2009; Hakamata et al., 2010; Wells et al., 2010).

## Conflict of interest statement

The authors declare that the research was conducted in the absence of any commercial or financial relationships that could be construed as a potential conflict of interest.
